# Air pollution data in COVID-19 time: A call for improving availability and accessibility

**DOI:** 10.7189/jogh.11.03089

**Published:** 2021-07-24

**Authors:** Cleonilde Nascimento, Sheilla Oliveira, Helotonio Carvalho

**Affiliations:** 1 Department of Biophysics and Radiobiology, Biological Sciences Centre, Federal University of Pernambuco, Recife, Brazil; 2 Department of Immunology, Aggeu Magalhães Institute (IAM), Oswaldo Cruz Foundation (FIOCRUZ), Recife, Brazil

COVID-19 has changed the world since it was first discovered in Wuhan, China in December 2019. Many countries imposed lockdowns to reduce SARS-Cov-2 transmission and minimize the need for hospitalization and/or an ICU. Even though, by the end of June 2021, more than 181 million cases had been registered and more than 3.9 million people had died around in the world [1]. This health emergence gave rise to highly articulated systems of data collection in order to keep track of the advance of the disease in every country. One of the most used and cited of these systems was created and is maintained by Johns Hopkins University [1,2].

Lockdowns had a huge impact on people’s lives, making home-office the rule, closing schools, restaurants, and non-essential stores, and ultimately, saving millions of lives. The decrease in economic activities and transportation had a positive side effect, reducing air pollution in many cities/countries [3-5]. Air pollution causes more than 7 million deaths each year and particulate matter (PM) is the component most associated with health problems, such as ischaemic heart disease, lung cancer, cerebrovascular disease, chronic obstructive pulmonary disease and lower respiratory infections [6,7].

COVID-19 effects on air pollution levels raised a question about air quality data accessibility. The last update for the Air Quality Database from the World Health Organization (WHO) lists PM data for 4300 cities in 108 countries, comprising data from 9690 air quality monitoring stations [8]. Most stations are located in USA, Europe, Japan and China and the total number of stations and cities monitored in the world is, in fact higher comprising more than 15 000 air quality monitoring stations in over 6000 cities [9]. At least partly, the differences between the numbers in these two reports may reflect data accessibility. Access to historical air pollution data varies widely and many cities around the world do not make data readily available. For those cities which have historical air pollution data and make them available, the way they are provided, and their accessibility is highly variable. Table 1 compares air quality data availability for 23 selected cities, in different regions of the world and different levels of air pollution. Islamabad has no city-specific website and the national one has only limited information. For Dubai, anyone who wants to access the data needs to make a payment to the Dubai Municipality. Each pollutant is charged separately and the longer the time frame for the data the higher is the fee amount. For PM_2.5_ (particulate matter below 2.5 μm in diameter), for example, each month of data costs about US$400.00.

**Table 1:** Comparison of air quality data accessibility in different cities of the world

City/PM_2.5_ annual mean (μg/m^3^)*	Live data/ historical data sets	Data sets for individual stations	Search for all stations/ pollutants at once	Time frame availability (PM_2.5_)†	Restriction for time frame search	Page in English/data search	Additional features/issues	Website
Adelaide (7)	Yes/Yes (csv files)	Yes	No	2013-To date	None	NA		https://www.epa.sa.gov.au
Auckland (6)	Yes/Yes (csv files)	Yes	No/No	Not evaluated	None	NA	Website not user friendly	https://environmentauckland.org.nz
Beijing (73)	Yes/Not evaluated	Not evaluated	Not evaluated	Not evaluated	Not evaluated	No/NA	Website cannot be translated	http://zx.bjmemc.com.cn
Berlin (16)	Yes/Yes (csv files)	Yes	No/Yes	2016-To date	1 y at a time	Yes/No	Data in graphs	https://luftdaten.berlin.de/lqi
Buenos Aires (N/A)	No/Yes (pdf files)	Yes	No/Yes	2010-To date (PM_10_). No PM_2.5_ data	1 mo at a time	No	2-mo delay on reports	https://www.buenosaires.gob.ar/agenciaambiental
Dubai (54)	Yes/Upon payment	Not evaluated	Not evaluated	Not evaluated	Not evaluated	Yes/NA		http://www.dubaiairenvironment.dm.gov.ae
Islamabad (66)	No/No	NA	NA	NA	NA	Yes/No		http://www.environment.gov.pk/index.php
Johannesburg (41)	Yes (AQI)/Yes (xls files)	Yes	Yes/Yes	2016-To date	None	NA	Lack of continuous data	https://saaqis.environment.gov.za/home/index
London (12)	Yes/Yes (csv files)	Yes	Up to 6 stations/No	2009-To date	None	NA	Data in graphs; different times (15’-24 h)	https://www.londonair.org.uk
Los Angeles (12)	Yes/Yes (csv files)	Yes	Yes/No	1999-To date	1 y at a time	NA		https://ww2.arb.ca.gov
Mexico City (22)	Yes/Yes (csv files)	Data sets contain all stations	Yes/Yes	2003-To date	None	No/NA		http://www.aire.cdmx.gob.mx
Milan (27)	Yes/Yes (csv files)	Yes	No/5 pollutants at a time	2007-To date	1 y at a time	No/NA	Data sent by email; search is troublesome	https://www.arpalombardia.it
Moscow (14)	Yes/Yes (csv files).	Data sets contain all stations	Yes/Yes	Not evaluated	None	Yes/NA	Data in graphs; search is troublesome.	https://mosecom.mos.ru (live data); https://data.mos.ru/ (historical data)
New Delhi (143)	Yes/Yes (csv files)	Yes	Yes/Yes	Not evaluated	None	Yes/Yes	Data at different times (15min-24 h)	https://app.cpcbccr.com
New York (7)	Yes/Yes (xls files)	Yes	Yes	2015-To date	None	NA	Older data: US EPA website	http://www.nyaqinow.net; https://www.epa.gov/outdoor-air-quality-data (older data)
Paris (16)	Yes/Yes (csv files)	Data sets contain all stations	Yes, download station or pollutant data	1999-To date	None	No/No		https://www.airparif.asso.fr
Rome (15)	Yes/Yes (pdf files)	Only for real-time data	Yes/No	1999-To date	1 y at a time	Yes/NA	Search is troublesome	http://www.arpalazio.gov.it
Santiago (29)	Yes/Yes (csv files)	Yes	No/No downloaded separately	1997-To date	None	No	Data in graphs	https://sinca.mma.gob.cl
São Paulo (17)	Yes/Yes (csv files)	Yes	1 station/3 pollutants a time	Not evaluated	1 y at a time	No	Requires registration	https://qualar.cetesb.sp.gov.br
Seoul (26)	Yes/Yes (CSV files), but in Korean	Yes	No/Yes	2013-To date	2 mo at a time	Yes/No	Hard to navigate; search is troublesome	https://www.airkorea.or.kr
Sydney (8)	Yes/Yes (csv files)	Yes	Yes/Yes	2012-To date	None	NA		https://www.dpie.nsw.gov.au
Tokyo (17)	Yes/Yes but in Japanese	Not evaluated	Not evaluated	2000-To date	None	Yes/No	6-mo delay on reports; website cannot be translated	https://www.kankyo.metro.tokyo.lg.jp
Venice (26)	Yes/Only daily bulletins	Yes	Yes	2010-To date	Not evaluated	No/NA	Data upon request (csv files)	https://www.arpa.veneto.it

N/A – not applicable, y – year, mo – months

*PM_2.5_ data from WHO Global Ambient Air Quality Database, 2018, https://www.who.int/airpollution/data/cities/en/, except for Dubai, which was calculated from [8].

†Time frame availability was analysed for PM_2.5_ data. Data for other pollutants usually have larger time coverage.

Time frame availability, evaluated for PM_2.5_, varies widely. While some cities have data since the late 1990s, others have only after 2010s. For Berlin, many air quality monitoring stations started measuring PM_2.5_ in 2008, but only data from 2016-To date are available, probably a temporary issue in order to revise older data. Buenos Aires has no PM_2.5_ historical data, while they are discontinuous for Johannesburg. Data output format can also be a problem. This is the case for Buenos Aires and Rome, since data are only available as reports in pdf files, instead of Excel or CSV, which makes it more difficult to work with them.

In some cases, the absence of an English webpage or its limited functionality is a real problem. This issue can sometimes be overcome with automatic translation functions available in internet browsers. However, it is often impossible to access air pollution data. This is the case for Beijing. The information is apparently there, but translation engines are not able to translate them. In this case, websites that mirror some air pollution data in China and can be automatically translated, such as China's air quality online monitoring and analysis platform-AQI [10] may be a way to get some data. Japan is the country with the largest coverage of air quality stations per area [9]. However, Tokyo air quality website makes it virtually impossible to access the data without knowing Japanese. There is an English webpage but with only limited functions. For Seoul, the English webpage has no data search function. The Korean website, when accessed by browser translation, is difficult to navigate and find the data, especially for individual air monitoring stations. For Moscow, interestingly, current year data can be visualized in graphs for each air quality monitoring stations and air pollutant but cannot be downloaded. Data download is possible in another website which has an English webpage. However, search is troublesome, and data are downloaded partially in English and partially in Russian.

Despite the absence of English versions, some websites work well with browser translation and may be accessible for people who do not speak the original language. This is the case for Venice, Milan, Rome, Madrid, Mexico City, Buenos Aires, and São Paulo. Berlin has an English webpage but with no data search function though the German website can be translated enabling data search. For Paris, the website available until early 2021, had an English webpage with the same functions as the French website. Unfortunately, the new website does not have an English version anymore. Finally, São Paulo is the only city which requires registration to access the data. As examples of data availability and accessibility, it is noteworthy to mention London and New Delhi websites which are easy to navigate and allow download of data with different time intervals from 15 minutes to 24 hours.

**Figure:**
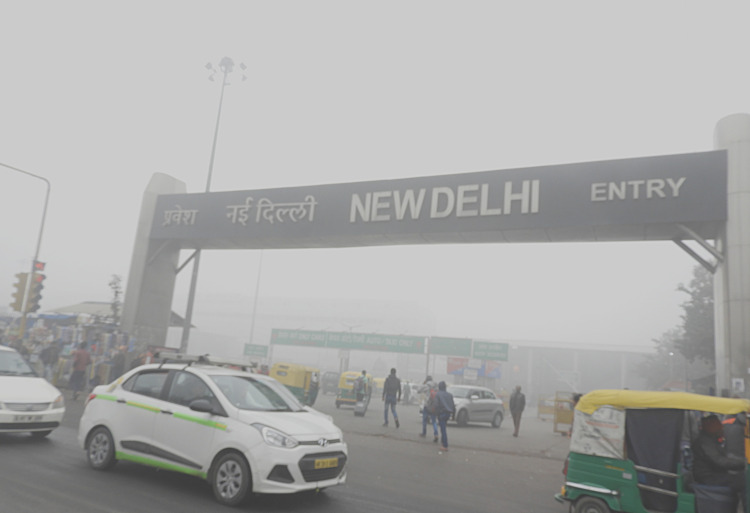
Photo: Low visibility due to smog at New Delhi railway station (Sumita Roy Dutta, via Wikimedia Commons).

The purpose here was to compare air pollution data accessibility in different cities around the world. Despite some good examples, many cities need to improve data accessibility. Seoul, Tokyo, and Beijing, some of the cities with major problems in air pollution data accessibility due to language issues, would benefit from the experience from cities like London and New Delhi. Islamabad, one of the most polluted capitals in the world, should give priority to improving data transparency. Besides Islamabad, many other highly polluted cities in Southeast Asia or the Middle East do not have any record of official air quality monitoring. These include Peshawar, another Pakistan city, Ulaanbaatar, Kabul, Manama, and Kathmandu. For all of them, except Kabul, US Embassies and Consulates are a helpful alternative data source since they have their own air quality monitoring [11]. Other agencies like US Environmental Protection Agency (EPA) and European Environmental Agency (EEA) provide data access for several US and European cities, respectively [12,13]. Despite their value, it is not always possible to search for specific air quality monitoring stations or identify them.

Air quality depends on monitoring, data availability, and policies to decrease air pollution levels, which help to reduce health problems and deaths caused by air pollution. Changes in air pollution, caused by COVID-19 pandemics, brought air quality to attention of the public and governments, which should be used to speed up the adaptation of agencies like US EPA and EEA to allow more detailed air quality data search. Besides this, it should also induce cities/countries to improve transparency and provide English webpages with full data access. Finally, a global service which would be updated in real-time with historical data from air quality monitoring stations from all over the world, like the Johns Hopkins University website for COVID-19, would be a really useful tool, which would greatly increase transparency and accessibility to air quality data.
